# Polymorphisms in genes *TLR1*, *2* and *4* are associated with differential cytokine and chemokine serum production in patients with leprosy

**DOI:** 10.1590/0074-02760160366

**Published:** 2017-03-02

**Authors:** Nadja de Lima Santana, Jamile Leão Rêgo, Joyce Moura Oliveira, Lucas Frederico de Almeida, Marcos Braz, Lídia Maria Medeiros Machado, Paulo Roberto Lima Machado, Léa Cristina Castellucci

**Affiliations:** 1 Instituto Nacional de Ciência e Tecnologia em Doenças Tropicais, Salvador, BA, Brasil; 2 Universidade Federal da Bahia, Hospital Universitário Professor Edgard Santos, Serviço de Imunologia, Salvador, BA, Brasil; 3 Universidade Federal da Bahia, Programa de Pós-Graduação em Ciências da Saúde, Salvador, BA, Brasil

**Keywords:** TLR, polymorphism analysis, cytokine and chemokine serum production, leprosy

## Abstract

**BACKGROUND:**

Leprosy or hansen’s disease is a spectral disease whose clinical forms mostly depends on host’s immune and genetic factors. Different Toll-like receptors (TLR) variants have been described associated with leprosy, but with some lack of replication across different populations.

**OBJECTIVES:**

To evaluate the role of polymorphisms in genes *TLR1*, *TLR2* and *TLR4* and susceptibility to leprosy in a genetic case control study; to verify the association between genotypes of these markers and the immunological profile in the serum of patients with leprosy.

**METHODS:**

Pre-designed TaqMan® assays were used to genotype markers at *TLR1* (rs4833095, rs5743551), *TLR2* (rs7656411, rs3804099) and *TLR4* (rs1927914, rs1927911). A panel of cytokines and chemokines was accessed by enzime-linked immunosorbent assay (ELISA) test in the serum of a subgroup of patients with and without leprosy reactions.

**FINDINGS:**

Our results show an association between the T allele of rs3804099 at the *TLR2* gene and increased risk for leprosy per se [Odds ratio (OR) = 1.296, p = 0,022]. In addition, evaluating the association between different genotypes of the TLR1, 2 and 4 markers and cytokine/chemokine serological levels, IL-17 appears as an immunological marker regulated by the polymorphism of the three TLR genes evaluated, whereas different *TLR1* genotypes were associated with differential production of IL-12p40 and MCP-1(CCL2). Furthermore, other relevant serum markers such as CXCL-10 and IL-6 seemed to be regulated by *TLR2* variants and IL-1β was related to *TLR4* genotypes.

**MAIN CONCLUSIONS:**

All together our data points that the tested TLR markers may have a regulatory role in the immunity against *Mycobacterium leprae,* by driving the host’s production of key cytokines and chemokines involved in the pathogenesis of this disease.

Leprosy is a chronic infectious disease caused by the bacillus *Mycobacterium leprae* that mainly affects the skin, peripheral nerves, upper respiratory tract mucosa and eyes (Scollard et al. 2006). The most probable mode of transmission of this disease is through prolonged contact with nasal and mouth secretions and infected skin of contagious subjects. Leprosy is a spectral disease classified according to the type and gradation of the host immunity. Thus, patients with tuberculoid leprosy (TT) are characterised by a relevant cellular immune response, manifested by few cutaneous or neural lesions with little or no bacilli and a TH1 cytokine profile. In contrast, patients with lepromatous leprosy (LL) are in an opposite pole, characterised by multiple lesions, high bacterial load, diminished or absent lymphocyte proliferation and a TH2 cytokine response (Ramos et al. 1989). The polar groups TT and LL are stable, but the Borderline forms through the spectrum are characterised by unstable immune response and may present progressive reduction of cell-mediated immunity (Ridley & Jopling 1966). In addition, throughout the natural course of the disease, patients may also develop acute inflammatory episodes namely leprosy reactions, that are classified as type 1 (reverse reaction - RR) or 2 (erythema nodosum leprosum - ENL). Leprosy reactions are due to immune inflammatory responses against *M. leprae* antigens in the peripheral nerves and skin, with several clinical manifestations. Although clinically different, both reaction types can lead to nerve damage and evolve with physical disabilities (Cardoso et al. 2011). Leprosy is influenced by host genetic factors and environmental factors such as nutritional status, BCG vaccination and exposure rate to *M. leprae* or other mycobacteria (Moraes et al. 2006). The low genetic variability of the bacilli reinforces the importance of the host genetic background as a prominent factor for development of disease upon infection. Several studies have shown the influence of gene variants in the development of leprosy *per se* or its clinical forms (Mira et al. 2004, Cardoso et al. 2011, Zhang et al. 2011, Fava et al. 2012, Liu et al. 2013).

The ten-member Toll-like receptor Family (TLRs) recognises particular molecular patterns of diverse microorganisms in early innate immune responses, being considered important adaptors in the host-pathogen interaction. TLRs are expressed by a variety of leukocytes and solid tissue cell types, with the highest levels primarily displayed by cells of myeloid lineage such as monocytes, macrophages, and dendritic cells (Hart & Tapping 2012). The contribution of the TLR variations to the susceptibility for leprosy has been investigated in different populations, and *TLR1*, *TLR2* and *TLR4* variants have been reported to be associated with leprosy by previous studies in ethnical different populations such as Indian, Nepalese, African and Brazilian (Johnson et al. 2007, Bochud et al. 2008, 2009, Misch et al. 2008, Hart & Tapping 2012, Marques et al. 2013). In particular, polymorphisms located at *TLRs* genes were repeatedly associated with leprosy *per se* and leprosy reactions (Bochud et al. 2008, Schuring et al. 2009, Wong et al. 2010)[Bochud, 2008 #20;Schuring, 2009 #21;Wong, #22;Sardinha, 2011 #24].

In this study we show that a polymorphism at *TLR2* (rs3804099) previously associated with leprosy type 1 reaction, was positively associated with increased risk for developing leprosy *per se* in a population from Northeast Brazil. Additionally, there were an association between carriage different alleles at genes *TLR1*, *2* and *4* and serum level of cytokines and chemokines across leprosy spectrum.

## MATERIALS AND METHODS

*Case patients, control subjects, and study design* - The study participants were enrolled from two reference centres in the city of Salvador, Bahia (BA), Brazil (Hospital Universitário Professor Edgard Santos and Hospital Couto Maia). Leprosy patients were enrolled after diagnostic confirmation by dermatological and neurological evaluation, sensitivity test, bacillary index and histopathology of one or more skin lesions to classify by Ridley and Jopling (1966) criteria the clinical form of the disease. Three hundred and sixty-two leprosy cases of both genders, aging 18 to 65 years under multidrug therapy and monthly follow-up were included as cases. The control group consisted of 368 individuals recruited as volunteer blood bank donors in the city of Salvador (HEMOBA Foundation). All controls were inquired about personal or family history of leprosy prior sample collection. The characteristics of the patients cohort are described elsewhere (Rego et al. 2015). A subset of 52 leprosy subjects was used for the enzime-linked immunosorbent assay (ELISA) tests, providing a sample of 17 without reaction (10 PB; 7 MB) and 35 with reactions (22 with type I reaction/RR; 13 with type II reaction/ENL). The subjects with reactions were free of immunosuppressive drugs such as prednisone and thalidomide (in case of type II reactions) when the serum sample was collected. Informed consent was obtained from all participants. Approval for the use of the samples in this study was obtained from the Federal University of Bahia (CEP-891.963) and the Brazilian National Ethical Committee (CONEP-759/2010).

*DNA extraction and genotyping* - DNA was obtained from all samples by blood venipuncture and collected into dodecyl citrate acid- containing Vacutainers (Becton Dickinson). Genomic DNA was prepared using the proteinase K and *salting-out* method previously described (Sambrook et al. 1989). Validated predesigned Taqman® qPCR assays, containing polymerase chain reaction (PCR) primers and probes were purchased from Life Technologies® (Thermo Fisher, Inc) and reactions prepared according to the manufacturer’s protocols. The genotyped single nucleotide polymorphisms (SNPs) were chosen based on literature data coupled with allele frequencies considering both, Caucasian and Youruba populations available in the 1000 Genomes project as follows: rs4833095 (N248S) and 5743551 for the *TLR1* gene; rs3804099 and 7656411 for the *TLR2* gene; rs1927914 and rs1927911 for the *TLR4* gene. Additional details regarding the markers are shown on Table I. To ensure the accuracy of genotyping results, three positive controls and a negative control were included in each 96-well plate. Taqman® assays were performed using the 7500 standard (Life Technologies), and the ABI software v 2.0.6 was used to analyse the data.

**TABLE I:** Details of the *TLR1*, *2* and *4* genotyped single nucleotide polymorphisms as recorded in 1000 Genomes, GRCh37 assembly

GENE/SNP	Chrossome: physical position	Alleles	Variation type
TLR1			
rs4833095	4: 38799710	C/T	Missense variant
rs5743551	4: 38807654	C/T	Intron variant
TLR2			
rs3804099	4: 154624656	C/T	Synonymous variant
rs7656411	4: 154627655	G/T	Downstream gene variant
TLR4			
rs1927914	9: 120464725	A/G	Upstream gene variant
rs1927911	9: 120470054	A/G	Intron variant

TLR: Toll-like receptors.

*ELISA cytokine assays* - Whole blood was collected by venipuncture and centrifuged at 20,000g for 10 min for serum obtaining. Levels of the cytokines IL-1β, IL-6, IFN-g and IL12p40, IL-17, IL-10 were measured in serum using commercial kits from R&D (R&D systems Inc. Minneapolis, MN, US) and BD OptEIA™ Set human (BD Biosciences, San Jose, CA, US), respectively, according to manufacturer’s protocols. To measure the TNF levels, a high sensitivity ELISA sandwich technique was used (NOVEX®, Termo Fisher, Inc), also following the manufacturer’s instructions. Optical density was measured in the spectrophotometer at 450 nm. The results were expressed in pg/mL, based on comparisons with standard curves for each cytokine kit.

*ELISA chemokine assays* - Levels of the chemokines IL-8, MIP-1α, MIP-1β and MCP-1, CXCL-9, CXCL-10 were measured in serum using commercial kits from R&D (R&D systems Inc. Minneapolis, MN, US) and BD OptEIA™ Set human (BD Biosciences, San Jose, CA, US), respectively, according to manufacturer’s protocols. Optical density was measured in the spectrophotometer at 450 nm. The results were expressed in pg/mL, based on comparisons with standard curves for each chemokine kit.

*Statistical analysis* - Unconditional logistic regression analysis was performed using STATA (version 8.2; available from: http:// www.stata.com/) with the freely available GenAssoc package (available from: http://www-gene.cimr.cam.ac.uk/clayton/software/stata/) to determine allele-wise (1 *df* test) and genotype-wise (2 *df* test) associations comparing cases and controls. Global test statistics were generated for both the 1 *df* test and the 2 *df* test, and odds ratios (ORs) with 95% confidence intervals (CIs) were calculated. Analysis of Hardy-Weinberg equilibrium (HWE) was carry out considering unrelated and unaffected individuals. The ELISA results were analysed using the software programs Instat3 and GraphPadPrism5. The comparison of two independent groups was performed using the Mann-Whitney test, whereas for statistical comparison of more independent groups the One-way ANOVA Kruskal-Wallis test was used. Differences were considered statistically significant when the p value was below 0.05 (p ˂ 0.05).

*Ethics* - This study was approved by the ethical committee of the Faculdade de Medicina da Universidade Federal da Bahia (Nº-891.963) and Comissão Nacional de Ética em Pesquisa - CONEP (Nº-759/2010).

## RESULTS

*Population-based analysis of the genes TLR 1, 2 and 4* - Table II provides details about allelic and genotypic frequencies among cases and controls for the markers genotyped. All SNPs were in Hardy Weinberg equilibrium (p > 0.05, data not showed). We found no association between markers at *TLR1* (rs4833095, namely N248S, and rs5743551) and *TLR4* (rs1927914 and rs1927911) and leprosy *per se* or leprosy reactions in this population (p ˃ 0,05). Results of the unconditional logistic regression analysis for these phenotypes are presented in Table III. On the other hand, there was a significant association between the T allele of the rs3804099 marker and susceptibility with the leprosy *per se* status (OR = 1.29; CI = 1.03-1.62; global p = 0.021). There was no association between disease and the other marker at *TLR2*, rs7656411, Table II.

**TABLE II:** Allele and genotype frequencies of the *TLR1*, *2* and *4* markers between cases and controls

Gene/marker	Allele/genotype	Cases n(%)	Controls n(%)	Total (%)
*TLR1*_rs4833095 C/T				
	C	382(58,8%)	407(61,9%)	789(60,3%)
	T	268(41,2%)	251(438,1%)	519(39,7%)
	CC	117(36,0%)	122(37,1%)	239(36,5%)
	CT	148(45,5%)	163(49,5%)	311(47,6%)
	TT	60(18,5%)	44(13,4%)	104(15,9%)
*TLR1*_rs5743551 C/T				
	C	387(59,2%)	410(61,9%)	797(60,6%)
	T	267(40,8%)	252(38,1%)	519(39,4%)
	CC	121(37,0%)	127(38,4%)	248(37,7%)
	CT	145(44,3%)	156(47,1%)	301(45,7%)
	TT	61(18,7%)	48(14,5%)	109(16,6%)
*TLR2*_rs3804099 C/T				
	C	325(50,3%)	372(56,5%)	697(53,5%)
	T	321(49,7%)	286(43,5%)	607(46,5%)
	CC	76(23,5%)	105(31,9%)	181(27,8%)
	CT	173(53,6%)	162(49,2%)	335(51,4%)
	TT	74(22,9%)	62(18,8%)	136(20,9%)
*TLR2*_rs7656411 G/T				
	G	269(44,1%)	267(44,4%)	536(44,2%)
	T	341(55,9%)	335(55,6%)	676(55,8%)
	GG	57(18,7%)	56(18,6%)	183(30,2%)
	GT	155(50,8%)	155(51,5%)	310(51,2%)
	TT	93(30,5%)	90(29,9%)	113(18,6%)
*TLR4*_rs1927911 A/G				
	A	269(40,4%)	269(40,4%)	478(40,9%)
	G	397(59,6%)	397(59,6%)	690(59,1%)
	AA	52(15,6%)	52(15,6%)	212(36,3%)
	AG	165(49,5%)	165(49,5%)	266(45,5%)
	GG	116(34,8%)	116(34,8%)	106(18,2%)
*TLR4*_rs1927914 A/G				
	A	337(50,9%)	223(46,5%)	560(49,0%)
	G	325(49,1%)	257(53,5%)	582(51,0%)
	AA	89(26,9%)	59(24,6%)	148(25,9%)
	AG	159(48,0%)	105(43,8%)	264(46,2%)
	GG	83(25,1%)	76(31,7%)	159(27,8%)

TLR: Toll-like receptors.

**TABLE III:** Results of logistic regression analyses for the genotyped Toll-like receptors (TLR) polymorphisms

Cases *Vs*. Controls			
*TLR1*_rs4833095	Odds ratio (OR)	Confidence interval (CI 95%)	p value
C	0,879	0,70-1,09	0,256
T	1,136	0,91-1,41	0,256
C/T X T/T	0,665	0,42-1,04	0,075
C/C X T/T	0,703	0,44-1,11	0,137
			
*TLR1*_rs5743551	OR	CI (95%)	p value
C	0,894	0,72-1,11	0,316
T	1,117	0,89-1,38	0,316
CT X T/T	0,731	0,47-1,13	0,164
C/C X T/T	0,749	0,47-1,17	0,212
			
*TLR2*_rs3804099	OR	CI (95%)	p value
C	0,771	0,61-0,96	0,022
T	1,296	1,03-1,62	0,022
C/T X T/T	0,894	0,59-1,33	0,585
C/C X T/T	0,606	0,38-0,94	0,029
			
*TLR2*_rs7656411	OR	CI (95%)	p value
G	0,989	0,78-1,24	0,928
T	1,01	0,80-1,27	0,928
GT X T/T	0,967	0,67-1,39	0,86
G/G X T/T	0,985	0,61-1,57	0,95
			
*TLR4*_rs1927911	OR	CI (95%)	p value
A	0,952	0,75-1,19	0,678
G	1,049	0,83-1,31	0,678
A/G X G/G	1,351	0,93-1,95	0,107
A/A X G/G	0,796	0,49-1,27	0,341
			
*TLR4*_rs1927914	OR	CI (95%)	p value
A	1,18	0,94-1,48	0,153
G	0,847	0,67-1,06	0,153
A/G X G/G	1,386	0,93-2,06	0,107
A/A X G/G	1,381	0,87-2,17	0,162

**Fig 1:**
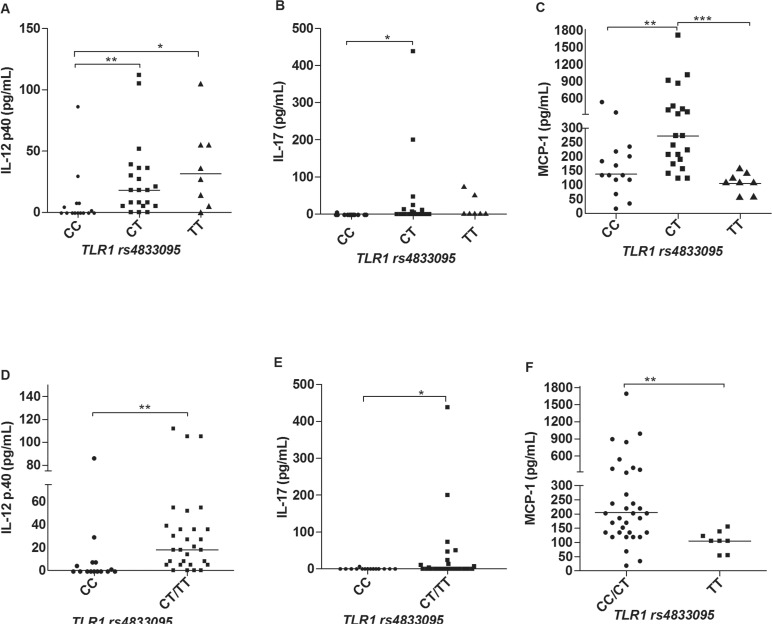
: serum levels of IL-12p40 (A, D), IL-17 (B, E) and MCP-1 (C, F) across different *TLR1* rs4833095 genotypes. The non-parametric tests of Kruskal-Wallis and Mann-Whitney were used to analyse the statistical differences (N = 52). *p < 0.05; **p < 0.01; ***p < 0.001.

**Fig. 2:**
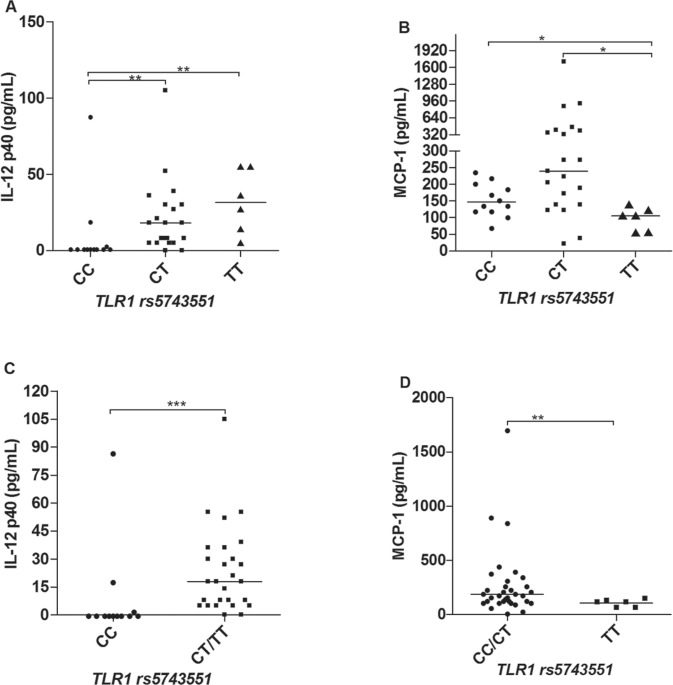
: serum levels of IL-12p40 (A, C) and MCP-1 (B, D) comparing different *TLR1* rs5743551 genotypes. The non-parametric tests of Kruskal-Wallis and Mann-Whitney were used to analyse the statistical differences (N = 52). *p < 0.05; **p < 0.01; ***p < 0.001.

*Cytokine and chemokine profiles in leprosy patients according to different genotypes of TLR1 SNPs* - There were differences in the serum levels of some cytokines and chemokines regarding the *TLR1* polymorphisms. For the SNP rs4833095 (genotypes CC, CT and TT) we observed significant differences in the IL-12p40 and IL-17 levels, in which patients carriers of the T allele produced higher amounts of these two cytokines in comparison to individuals homozygous for the CC genotype (Fig. 1A-B), respectively. These results become more evident by pooling together the genotypes TC and TT and comparing with the CC genotype, as shown in Fig. 1D-E. In addition, we also observed differences in the production of the chemokine MCP-1. In this case however, carriers of the TT genotype (TC and TT) produced lower serum levels of this chemokine as compared to CC and CT individuals, as shown in Fig. 1C, F. Regarding the *TLR1* marker, rs5743551 (genotypes CC, CT and TT), we also observed significant differences in relation to the production of IL-12p40 and MCP-1. Carriers of the T allele produced higher levels of IL-12p40 (Fig. 2A, C), and, by the other hand, lower levels of MCP-1 (Fig. 2B, D). That accordance with the marker rs4833095 is expected, considering the linkage disequilibrium (LD) between these two SNPs that could account for similar results. There were no significant differences observed to other cytokines (IL-1β, IL-6, IFN-g and IL-10) and chemokines (IL-8, MIP-1α, MIP-1β, CXCL-9 and CXCL-10) evaluated in this study.

**Fig. 3:**
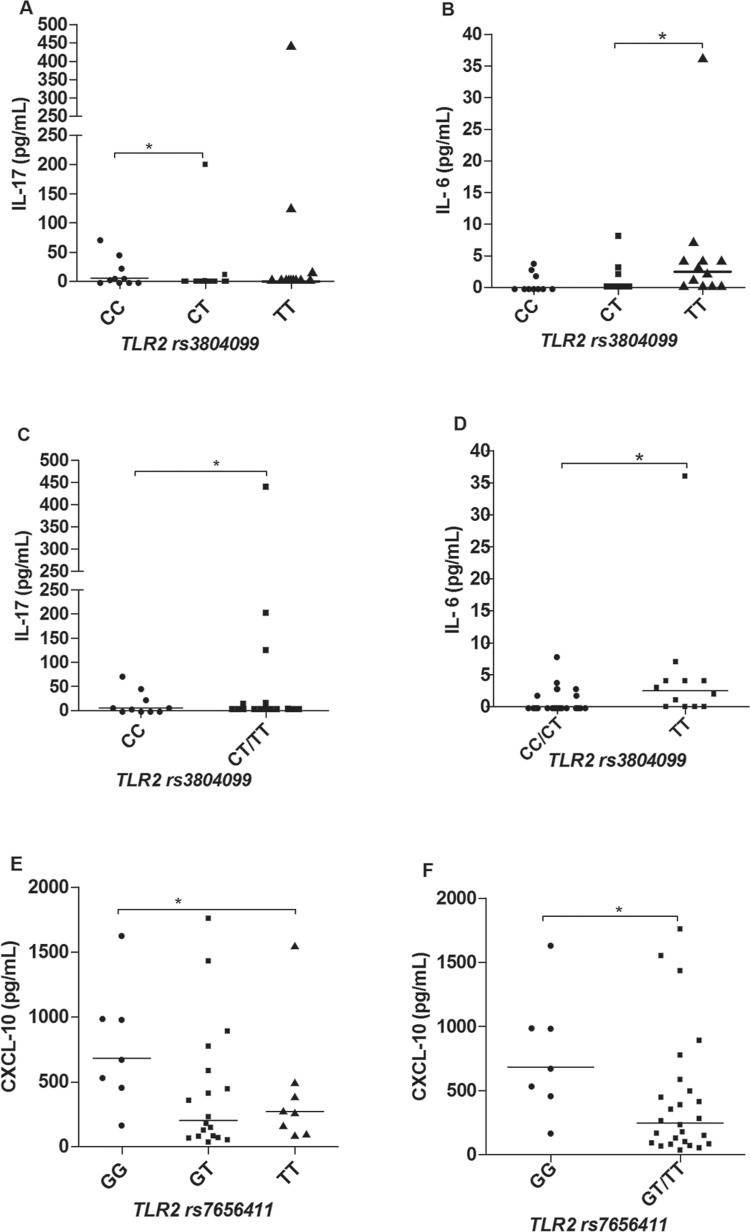
: serum levels of IL-17 (A, C) and IL-6 (B, D) according to different *TLR2* rs3804099 genotypes; and CXCL-10 across *TLR2* rs7656411 genotypes (E, F). The non-parametric tests of Kruskal-Wallis and Mann-Whitney were used to analyse the statistical differences (N = 52). *p < 0.05.

*Cytokine and chemokine profiles in leprosy patients according to different genotypes of TLR2 SNPs* - In the analysis of cytokines and chemokines production across the different genotypes of *TLR2* markers we found significant differences as described. For the rs3804099 (genotypes CC, CT and TT) carriers of the T allele produced higher serum levels of IL-17 (Fig. 3A) and this difference was kept significant by pooling together CT and TT individuals and comparing against CC individuals (Fig. 3C). We also observed that carriers of the T allele are higher producers of IL-6, especially when we join CT and CC and compare against the TT genotype (Fig. 3B, D). By the other hand, in relation to the marker rs7656411 (genotypes GG, GT and TT), we observed that carriers of the G allele produced higher levels of CXCL-10, as shown in (Fig. 3E-F). There were no significant differences observed to other cytokines and chemokines evaluated.

**Fig. 4:**
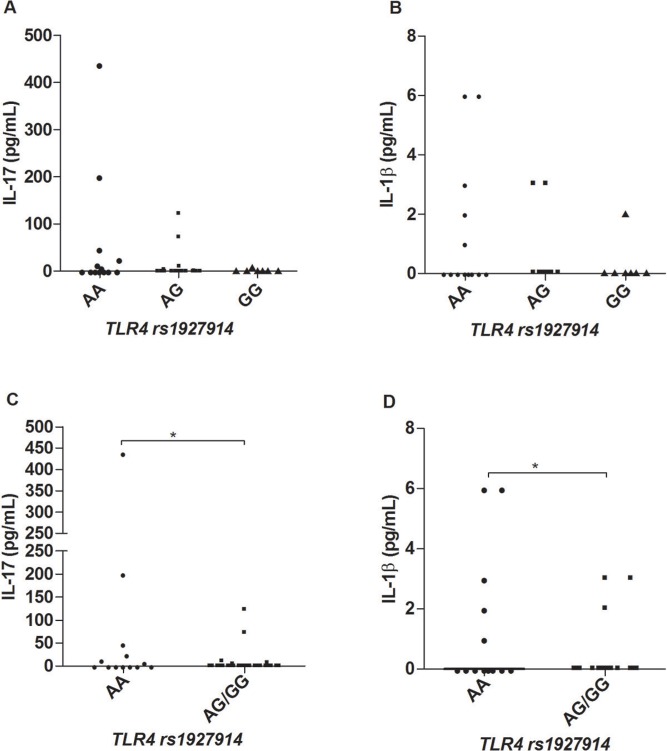
: serum levels of IL-17 (A, C) and IL-1β (B, D) according to different *TLR4* rs1927914 genotypes. Kruskal-Wallis and Mann-Whitney tests were used to analyse the statistical differences (N = 52). *p < 0.05.

*Cytokine and chemokine profiles in leprosy patients according to different genotypes of TLR4 SNPs* - We found no differences concerning cytokines or chemokines production and the different genotypes of the marker rs1927911 (genotypes AA, AG and GG), p > 0,05 (data not shown). For the marker rs1927914 (genotypes AA, AG and GG), we observed significant differences in the levels of the cytokines IL-17 and IL-1β, in which carriers of the A allele produced more of both cytokines as compared to carriers of the allele G allele (Fig. 4A-B), respectively. This difference become clearer when AA subjects were compared to subjects carrying the genotypes AG or GG together as shown in Fig. 4C-D.

## DISCUSSION

The broad spectrum of clinical and pathological manifestations of leprosy, aligned to its epidemiological, geographical and ethnic heterogeneity, greatly depend on the host genetic variability (Alter et al. 2008). Whereas some locus affect the intrinsic susceptibility to leprosy (leprosy *per se*), others modulate risk factors for the pauci or multibacillary forms of disease or the development of leprosy reactions (Mira et al. 2004). Interactions between bacterial, fungal and viral components and TLRs, activate the pathway of NF-kβ, driving the production of proinflammatory cytokine and chemokines, as well as the costimulatory molecules required for T-cell activation (Medzhitov et al. 1997, Taylor et al. 2012). The *M. leprae* and other species of mycobacteria such as *M. tuberculosis* are rich in various agonists for TLR family members including TLR1, 2 and 4 (Hart & Tapping 2012). In addition, previous studies had shown associations between variations at TLR genes and increased risk for leprosy or leprosy reactions. In this study, we observed an association between the T allele of the marker rs3804099 at the *TLR2* gene and susceptibility to leprosy *per se* comparing cases and controls. This polymorphism has been previously associated with the development of type I reactions in an Ethiopian cohort (Bochud et al. 2008). We stratified our samples regarding type I or type II reactions in the genetic analyses. However, no significant results were found (data not shown). It is possible that the large number of patients excluded from the cohort in this analysis has lead us to loose power to detect any associations, considering that this marker might have only a small effect.

Additionally, in a Danish population, this marker was also associated with response to anti-TNF therapy in patients with inflammatory bowel disease (Bank et al. 2014). Data such as that are relevant as confirm previous genome wide studies that pointed a gene sharing between leprosy and inflammatory bowel disease (Bank et al. 2014). Regarding *TLR1* gene, Marques et al. (2013) documented a significant association between the S allele (N248S, rs48033095) with leprosy in different Brazilian populations, analysed both, separately and in meta-analysis. In our population, however, this association was not confirmed. Conflicting results in genetic studies can occur for different reasons. Our population has different allele frequencies for this marker as compared to the other Brazilians populations, which points to a diverse ethnical background. Also, the clinical forms, including the presence and number of patients with type 1 or 2 reactions, normally vary among the studied cohorts which might concur to different results. Given its biological significance however, this data does not exclude that other alleles or haplotypes in the *TLR1* gene could contribute for the disease susceptibility in our population.

In the analysis of cytokines and chemokines stratified according to genotypes we observed differences that point to a functional role for some markers. In the case of *TLR1*, significant changes for IL-17 and MCP-1were found for markers rs4833095 and rs5743551 and IL-12p40 for rs4833095. The latter SNP was associated with leprosy in Brazilian populations, corroborating previous data from Bangladesh (Schuring et al. 2009). As already said, although not associated with leprosy in our case-control study, the rs4833095 is a non-synonymous coding SNP so these results indicate that this marker might have a regulatory role in the production of these molecules under *M. leprae* infection. Regarding IL-17 in leprosy, results are contradictory, with some studies indicating poor production in serum and low expression in situ, whereas other studies have shown an increased expression in leprosy lesions (da Motta-Passos et al. 2012, Trombone et al. 2012). In our cohort, leprosy patients’s serum produced higher IL-17 concentrations as compared to health controls (p < 0.05, data nor shown). Recently an immunohistochemical study has shown that MCP-1 was present in leprosy-affected nerves being this cytokine also associated with excessive deposit of extracellular matrix (Medeiros et al. 2015), which might be related to nerve damage. In relation to IL12p40, PBMC cultures stimulated with *M. leprae* antigen shown increased production of this cytokine in cells of patients compared to controls, highlighting a mechanism in which IFN-g downregulates IL-10 by the induction of IL-12 (Libraty et al. 1997). The ways these SNPs are grouped into haplotypes, as well as the influence of epigenetic regulation are decisive in how and, in which moment, they can exert their effects on the immune response following infection. The SNP rs5743551 is intronic, therefore the results observed could be due to a regulatory effect from the marker itself or just by linkage disequilibrium with the rs4833095 or other functional marker.

Regarding SNPs in *TLR2* gene, we observed different concentrations of IL-6 and IL-17 related to the marker rs3804099. In this case, carriers of the T allele produced higher levels of these cytokines. This allele was also associated with increased risk of leprosy in our population. Considering that this marker is located in a coding region of the gene that strengthens the notion that it could have a functional role in the disease pathogenesis. IL-6 is a pro-inflammatory cytokine produced in high concentrations in leprosy and associated with the development of ENL (Stefani et al. 2009). The chemokine CXCL-10 appears differentially produced according to genotypes of the SNP rs7656411. Since this is an intronic SNP, we hypothesize that this could be the result of linkage disequilibrium with other regulatory markers. Regarding the *TLR4* gene, we observed greater production of IL-1-β and IL-17 between carriers of the A allele in the exonic SNP rs1927914. IL1-β is essential for the amplification of the T-cells specific immune response and its levels tend to decrease after the multidrug therapy (Moubasher et al. 1998). This cytokine is also produced in high concentrations in multibacillary patients (Madan et al. 2011). Finally, in order to check whether the association found is related to the disease or if it is a common effect seen in the general population, we analysed the immunological markers regarding the TLR genotypes in health controls (data not shown). Nonetheless, for most of the cytokines the values obtained in sera were zero or close to zero, making hard any type of comparison in this group. Regarding the chemokines, only for IL-8 we observed a borderline association between genotypes of *TLR2* rs7656411 (p = 0,059). That indicates that the polymorphisms are influencing the production of these immune parameters in the presence of infection.

Overall, our data indicates that different TLR genes may actually play a role in the IL-17 production. In addition, different markers of *TLR1*, *2* and *4* appeared associated with serum levels of other important cytokines and chemokines that take part in the leprosy pathogenesis. This data reinforces the regulatory role of genetic markers in infection and disease and highlights the premise that in multifactorial diseases various genes contribute to susceptibility or different clinical forms. Nevertheless, remains to be understood how this molecular network orchestrates a final phenotype. Much depends on the environment and this tight regulation must vary depending on personal stimuli.
